# Adiponectin preserves follicles through ADIPOR1/ADIPOR2-driven fatty acid metabolism

**DOI:** 10.1016/j.molmet.2026.102392

**Published:** 2026-06-10

**Authors:** Dae Hyun Lee, Jae Young Shin, Hyeri Park, Jin Seok, Kyu Hwan Na, Young-Ran Kim, Gi Jin Kim

**Affiliations:** 1 Department of Convergence Science, CHA University, 13488, Republic of Korea; 2 Reseach Institute of Placental Science, 13488, Republic of Korea; 3 PLABiologics Co., Ltd., 13488, Republic of Korea; 4 Department of Obstetrics and Gynecology, Fertility Center of CHA Bundang Medical Center, 13561, Republic of Korea

**Keywords:** Adiponectin, Mesenchymal stem cell, Metabolic syndrome, Fatty acid oxidation

## Abstract

**Background:**

Adiponectin is a key regulator of glucose and lipid metabolism that improves insulin sensitivity and promotes mitochondrial fatty acid oxidation via ADIPOR1 and ADIPOR2. Ovarian lipid accumulation contributes to metabolic reproductive disorders such as polycystic ovary syndrome (PCOS), yet current hormone-based therapies have limited efficacy and potential adverse effects. We evaluated whether placenta-derived mesenchymal stem cells (PDMSCs) mitigate ovarian lipotoxicity by restoring adiponectin signaling.

**Methods:**

A thioacetamide (TAA) induced rat model of metabolic dysfunction with ovarian lipotoxicity was treated by intravenous transplantation of PDMSCs (2 × 10^6^) cells. Hepatic and ovarian phenotypes were assessed four weeks after transplantation. In parallel, PDMSCs were cocultured with TAA-treated granulosa and primary theca cells, with or without siRNA-mediated knockdown of *ADIPOR1* and/or *ADIPOR2*.

**Results:**

PDMSCs transplantation improved systemic insulin resistance and dyslipidemia and partially restored hepatic and ovarian architecture. PDMSCs treatment increased circulating and ovarian ADIPONECTIN levels and upregulated *Adipor1/2* in ovarian tissue, accompanied by activation of the ADIPOR1/2/Fatty acid driven axis and enhanced mitochondrial fatty acid oxidation. These changes were associated with reduced ovarian lipid accumulation and improved endocrine homeostasis, including normalization of anti-Müllerian hormone (AMH), estradiol, and androgen levels, preservation of the primordial follicle pool, and induction of BMP15 expression. In vitro *ADIPOR1/2* silencing abrogated these protective effects, supporting a requirement for adiponectin receptor signaling.

**Conclusions:**

PDMSCs ameliorate systemic and ovarian metabolic dysfunction in a TAA-induced model, consistent with adiponectin, ADIPOR1/2 dependent mitochondrial metabolic reprogramming. PDMSCs restore both metabolic and reproductive competence in the context of hepatic-ovarian metabolic crosstalk. These findings support PDMSC-based therapy as a mechanistically informed, multi-target strategy for the treatment of PCOS and metabolic-associated ovarian dysfunction.

## Highlights

• PDMSCs transplantation restores both systemic and ovarian metabolic homeostasis in a TAA-induced metabolic dysfunction model.
• PDMSCs secrete adiponectin, reactivating the AdipoR1/2–PPARα–CPT1A signaling axis.
• Adiponectin receptor activation enhances mitochondrial fatty-acid oxidation and steroidogenic function.
• PDMSC therapy preserves follicular integrity by reducing atresia and maintaining ovarian reserve.
• Adiponectin signaling links lipid metabolic remodeling with restoration of ovarian function.

## 1 Introduction

Metabolic syndrome (MS) is characterized by insulin resistance, hypertension, and dyslipidemia, collectively increasing the risk of type 2 diabetes and cardiovascular disease [1]. In reproductive medicine, polycystic ovary syndrome (PCOS), which affects approximately 4–20% of women of reproductive age, is increasingly recognized as a metabolic–reproductive disorder due to its strong pathophysiological overlap with MS [2]. Insulin resistance and compensatory hyperinsulinemia drive ovarian androgen overproduction, lipid dysregulation, and inflammatory signaling, thereby exacerbating both metabolic and follicular dysfunction [3]. The multifactorial pathophysiology of PCOS involves complex interactions among genetic predisposition, insulin signaling abnormalities, mitochondrial dysfunction, inflammatory pathways, and endocrine imbalance [4].

Among the metabolic regulators implicated in PCOS, adiponectin has emerged as a critical adipocyte-derived hormone linking systemic metabolic homeostasis to ovarian function. Circulating adiponectin levels are consistently reduced in women with PCOS, independent of body mass index, and are inversely correlated with insulin resistance, hyperandrogenism, and dyslipidemia [5,6]. Adipose-derived extracellular vesicles have recently been identified as key mediators of inter-organ metabolic crosstalk, regulating insulin signaling and inflammatory responses in peripheral tissues [7]. Through binding to its receptors, AdipoR1 and AdipoR2, adiponectin activates AMP-activated protein kinase (AMPK) and peroxisome proliferator-activated receptor-α (PPARα) signaling pathways [8]. Activation of this axis enhances fatty acid oxidation, suppresses hepatic glucose production, improves lipid profiles by increasing high-density lipoprotein cholesterol (HDL-C) and reducing triglyceride levels [9], and mitigates oxidative stress and cardiometabolic risk [10,11]. Importantly, within the ovary, adiponectin promotes estradiol (E2) and progesterone synthesis while suppressing androgen production and regulating folliculogenesis, thereby directly linking hypoadiponectinemia to the pathogenesis of PCOS [12]. Moreover, adiponectin modulates glucose transporter expression, mitigates oxidative stress and cell cycle arrest, and promotes granulosa cell (GC) proliferation [11,13,14]. Therefore, dysregulation of the ADIPONECTIN–ADIPOR1/R2 axis may represent a central mechanism linking metabolic dysfunction to impaired ovarian steroidogenesis and follicular development in ovarian dysfunction. Despite the central role of metabolic dysregulation in ovarian dysfunction, current pharmacological treatments, including metformin and oral contraceptives, primarily target symptom control and do not fully restore metabolic homeostasis or inflammatory balance, and are often associated with adverse effects [15].

Stem cell-based interventions have been increasingly investigated as potential regenerative approaches for restoring ovarian function. Preclinical studies have reported improvements in hormonal balance, follicular development, granulosa cell survival, antioxidant capacity, and vascular remodeling following stem cell administration [16]. Although these findings suggest regenerative potential beyond conventional symptom-based therapies, most evidence currently derives from experimental and early clinical studies. Among the various stem cell types, MSCs have been widely studied because of their immunomodulatory and metabolic regulatory properties. Rather than acting primarily through direct tissue replacement, MSCs exert therapeutic effects mainly via paracrine signaling mechanism that modulates inflammation, enhances angiogenesis and supports tissue repair [17]. Studies employing bone marrow– and adipose-derived MSCs have demonstrated improvements in lipid metabolism, fatty acid oxidation, and insulin sensitivity, partly mediated by secreted bioactive factors such as adiponectin-related pathways, IL-10, and hepatocyte growth factor (HGF) [18].

Placenta-derived MSCs (PDMSCs) represent a particularly attractive cell source because of their high proliferative capacity, low immunogenicity, and noninvasive procurement [19]. In our previous studies, PDMSC transplantation improved hepatic lipid metabolism and enhanced ovarian glucose utilization in metabolic disease models, leading to restoration of follicular development and steroidogenic balance [20,21]. However, whether PDMSC therapy restores ovarian function through direct modulation of the of ADIPONECTIN–ADIPOR1/R2 signaling axis remains unclear.

In this study, we investigated whether intravenous transplantation of PDMSCs could ameliorate ovarian dysfunction in a rat model by reducing lipid accumulation, improving insulin sensitivity, and preserving follicular integrity. We further aimed to elucidate the underlying molecular mechanisms, focusing on activation of the ADIPONECTIN–ADIPOR1/R2 pathway and its role in regulating ovarian lipid metabolism and steroidogenesis.

## 2 Materials and methods

### 2.1 Stem cell culture

Placentas were obtained from women without any medical, obstetrical, or surgical complications who delivered at term (38 ± 2 gestational weeks). Placenta-derived MSCs (PDMSCs) were isolated from the chorionic plate of these normal-term placentas, following approval by the Institutional Review Board of CHA General Hospital, Seoul, Republic of Korea (CHAMC IRB-2025-06-037-002). PDMSCs were cultured in α-minimum essential medium (α-MEM; HyClone, USA) supplemented with 10% fetal bovine serum (FBS; Gibco, USA), 1% penicillin–streptomycin (Corning, USA), 25 ng/mL FGF-4 (PeproTech, USA), and 1 μg/mL heparin (Sigma–Aldrich, USA) [22]. All the cells were incubated at 37 °C with 5% CO_2_ and 95% humidity. For analysis of ADIPONECTIN secretion, WI38 fibroblast were cultured in α-MEM supplemented with 10% FBS and 1% P/S; BMMSCs were maintained in α-MEM supplemented with 10% FBS, 4 mM l-glutamine, and 1% P/S. All cell types were expanded to passage 7, seeded and cultured for 4 days under the above conditions, and one day prior to harvest, the medium was replaced with formulations containing serum-free α-MEM to minimize the influence of exogenous serum-derived factors.

### 2.2 Animal model establishment and PDMSC transplantation

Seven-week-old female Sprague–Dawley rats were obtained from KOATEC, Inc., Korea, and maintained in an air-conditioned facility. Metabolic dysregulation was induced by intraperitoneal (I.P.) injection of thioacetamide (TAA; Sigma–Aldrich, USA) at a dosage of 150 mg/kg, which was administered twice weekly for 12 weeks. Animals were randomly assigned to three groups (*n* = 5 per group): a normal control group (Nor), a TAA-injured nontransplantation group (NTx), and a TAA-injured group with PDMSC transplantation (PDMSC I.V). Intravenous (I.V.) transplantation of 2 × 10^6^ PDMSCs was performed at 8 weeks. The study concluded at 12 weeks post-TAA injection, at which time the animals were sacrificed. Ovary tissues and blood were harvested for blood chemistry analysis. Total cholesterol, high-density lipoprotein (HDL), low-density lipoprotein (LDL), and triglyceride (TG) levels were measured from the collected serum using reagents from DooYeol Biotech (Korea). Fasting glucose (mg/dL) and insulin (μU/mL) were measured after 12 h fast. Homeostatic model assessment of insulin resistance (HOMA-IR) = [fasting insulin × fasting glucose]/405; Homeostatic model assessment insulin sensitivity (HOMA-IS) = 1/HOMA-IR. Glucose was also expressed in mmol/L for internal quality control (QC) (conversion: mg/dL ÷ 18). All animal experimental procedures were conducted in accordance with the ethical guidelines and policies approved by the Institutional Animal Care and Use Committee (IACUC) of CHA University, Seongnam, Korea (IACUC-260003 approved on 2026.01.01).

### 2.3 Serum metabolomic analysis

#### 2.3.1 Sample preparation

Serum samples were thawed on ice prior to analysis. For metabolite extraction, 100 μL of serum was mixed with 100 μL of extraction solvent (acetonitrile:methanol = 4:1, v/v), vortexed for 5 min, and centrifuged at 13,000 rpm for 10 min at 4 °C. After centrifugation, 100 μL of the supernatant was transferred to LC–MS vials for analysis. QC samples were prepared by pooling equal aliquots of each sample.

#### 2.3.2 UPLC–QTOF–MS conditions

Metabolomic profiling was performed using an ACQUITY UPLC system (Waters, Milford, MA, USA) coupled to a Xevo G3 Q-TOF mass spectrometer (Waters) equipped with an electrospray ionization (ESI) source. Chromatographic separation was achieved on an ACQUITY UPLC HSS T3 column (2.1 × 100 mm, 1.8 μm). The mobile phases consisted of water with 0.1% formic acid (A) and acetonitrile with 0.1% formic acid (B), using the following gradient: 0–6 min, 5% B; 6–9 min, 5–95% B; 9–15 min, 95% B; 15–17.1 min, 95–5% B; and 17.1–20 min, 5% B. The flow rate was 0.3 mL/min, and the injection volume was 2 μL. Mass spectrometric data were acquired in both positive and negative ion modes under the following conditions: source temperature, 100 °C; desolvation temperature, 300 °C; desolvation gas flow, 600 L/h; cone gas flow, 50 L/h; and capillary voltage, 2.5 kV. The scan range was set to *m*/*z* 50–1200 with a scan time of 0.2 s. Leucine enkephalin (*m*/*z* 556.2771 [M+H] ^+^ and 554.2615 [M−H]^-^) was used as a lock mass for mass calibration. QC samples were injected at regular intervals throughout the analytical run.

#### 2.3.3 Data processing and statistical analysis

Raw LC–MS data were processed using Progenesis QI software (Nonlinear Dynamics, Newcastle, UK) for peak detection, alignment, and normalization. Features detected predominantly in blank samples were excluded, and QC-based filtering was applied to ensure analytical reproducibility. Metabolite annotation was performed by matching accurate mass, isotope distribution, and fragmentation spectra against public databases. Multivariate statistical analyses, including principal component analysis (PCA) and partial least squares–discriminant analysis (PLS-DA), were performed using SIMCA version 18.0 (Umetrics, Umeå, Sweden). Pathway enrichment analysis was conducted using annotated metabolites.

### 2.4 Gross morphological analysis and organ measurements

Gross morphological examination of liver and ovary tissues was performed for all experimental groups (Nor, NTx, and PDMSC I. V). After euthanasia, the liver and both ovaries were surgically harvested and immediately photographed using a digital camera (Canon, Japan) under standardized lighting and distance conditions. For consistency, images were captured with a fixed focal length, and a scale bar was included in each image for calibration. Organ weights were measured using an analytical balance, and values were normalized to body weight (g/g). To assess changes in size, the surface area of the ovaries was calculated from the captured images using ImageJ software (version 1.54 g, NIH, USA). The combined area of both ovaries was manually outlined, and the pixel count was converted to mm^2^ on the basis of the scale bar. In addition, the ovarian diameter was measured at the widest point using the same image processing software. All measurements were performed in triplicate and averaged for each animal.

### 2.5 Primary theca cell isolation and culture

Seven-week-old female Sprague–Dawley rats were euthanized via CO_2_ inhalation, and the ovaries were aseptically harvested. The excised ovaries were placed in McCoy’s 5A medium (Gibco, USA) supplemented with 2 mM l-glutamine (Sigma–Aldrich, USA) and 1% penicillin‒streptomycin (Gibco, USA). Surrounding fat was carefully removed, and theca cells were released into a sterile glass Petri dish by puncturing the follicles with a 26½-gauge needle. The remaining ovarian tissue was then transferred to a 100 μm cell strainer and enzymatically dissociated in McCoy’s 5A medium supplemented with 4 mg/mL collagenase (Sigma–Aldrich, USA), 50 ug/mL DNase I (Sigma–Aldrich, USA), and 10 mg/mL bovine serum albumin (BSA; RDT, USA) at 37 °C for 1 h, after which the samples were briefly vortexed. The digested cell suspension was filtered through a 40-μm cell strainer, centrifuged, and washed, and the isolated theca cells were cultured in McCoy’s 5A medium with supplements. Upon attachment, the cells were treated with 70 mM TAA or 100 μM Etomoxir (ETO, E1905, Sigma–Aldrich) for 24 h to induce cellular injury. Subsequently, the cells were cocultured with PDMSCs (6 × 10^4^ cells) for an additional 24 h using 8-μm pore-size Transwell inserts (Corning, USA), which allowed paracrine signaling without direct cell contact. All the cultures were maintained at 37 °C in a humidified incubator with 5% CO_2_.

### 2.6 Immortalized granulosa cells and RNA interference

KGN cells were provided by Riken Bio (Saitama, Japan). KGN cells were cultured in DMEM/F12 (GIBCO, USA) supplemented with 10% FBS (GIBCO) at 37 °C in a humidified atmosphere with 5% CO_2_. Upon attachment, the cells were treated with 70 mM TAA or 100 μM Etomoxir (ETO, E1905, Sigma–Aldrich) for 24 h to induce cellular injury. Subsequently, the cells were cocultured with PDMSCs (6 × 10^4^ cells) for an additional 24 h using 8-μm pore-size Transwell inserts (Corning), which allowed paracrine signaling without direct cell contact. siRNAs targeting AdipoR1 and AdipoR2 as well as mock siRNA were chemically synthesized (Bioneer, Korea) and transfected into 60–70% confluent KGN cells using Lipofectamine 2000 (Thermo Fisher Scientific, USA). The siRNA and Lipofectamine 2000 reagents were diluted and the cells were transfected according to the manufacturer’s protocol [23]. KGN cells were plated in 6-well plates and transfected with 5 μL of 20 μM stock siRNA. For the basal condition experiment, the transfected cells were cocultured with PDMSCs (6 × 10^4^ cells) for 24 h using 8 μm poresize Transwell inserts (Corning, USA) starting 24 h after transfection. In a separate set of experiments to validate the mechanism under metabolic stress, transfected cells were exposed to 70 mM TAA for 24 h to induce injury before being cocultured with PDMSCs for an additional 24 h.

### 2.7 Mitochondrial isolation

Mitochondria were isolated from frozen ovarian tissues using a Mitochondria Isolation Kit for Tissue (Thermo Fisher Scientific, USA) following the manufacturer’s instructions, with slight modifications [24]. Briefly, frozen tissue (50–100 mg) was homogenized in Reagent A, treated with Reagent B, and sequentially centrifuged (700×*g* then 12,000×*g*) to obtain a crude mitochondrial pellet. The resulting pellet was resuspended in Reagent C (wash buffer) and centrifuged once more at 12,000×*g* for 5 min to obtain the purified mitochondrial fraction. The final mitochondrial pellet was resuspended in storage buffer, and the protein concentration was subsequently determined using a BCA protein assay kit (Thermo Fisher Scientific, USA). Isolated mitochondria were either immediately used for downstream analysis or stored at −80 °C until further use.

### 2.8 Oxygen consumption rate assessment

Mitochondrial respiration was analyzed using a Seahorse XFe24 Extracellular Flux Analyzer (Agilent Technologies, USA) to evaluate oxidative metabolism and the fatty acid oxidation (FAO) capacity in KGN cells, theca cells, and PDMSC coculture models. Cells were seeded at a density of 2 × 10^4^ cells per well in Seahorse XF24 microplates and allowed to adhere overnight. Prior to measurement, the culture medium was replaced with Seahorse XF DMEM assay medium (5 mM glucose, 2 mM l-glutamine, 1 mM sodium pyruvate, and 5 mM HEPES, pH 7.4), and the plates were preequilibrated at 37 °C in a non-CO_2_ incubator for 30 min. The mitochondrial stress test was performed with sequential injections of oligomycin (1 μM), FCCP (2 μM), and rotenone/antimycin A (1 μM/2.5 μM), while the OCR was recorded in real time to calculate basal, ATP-linked, maximal, and nonmitochondrial respiration. To assess the FAO-dependent OCR, etomoxir (100 μM), an inhibitor of carnitine palmitoyl transferase 1a (CPT1A), was injected to block fatty acid transport into mitochondria. The FAO-specific OCR was calculated by subtracting the minimum OCR value after etomoxir treatment from the basal OCR prior to injection. All measurements were performed in triplicate and analyzed using Seahorse Wave software (v2.6.1.56).

### 2.9 RNA isolation and quantitative real-time polymerase chain reaction

Ovarian tissues, theca cells, and KGN cells were harvested and immediately snap frozen in liquid nitrogen. Total RNA was extracted using TRIzol reagent (Invitrogen, USA) according to the manufacturer’s instructions [21], purified by chloroform–isopropanol precipitation, washed with 70% ethanol, and dissolved in DEPC-treated water. RNA quality was assessed by NanoDrop, and cDNA was synthesized from total RNA using SuperScript III reverse transcriptase under the following standard thermal cycling conditions: 65 °C for 5 min, 4 °C for 1 min, 50 °C for 60 min, and 72 °C for 15 min. Quantitative real-time PCR (qRT‒PCR) was performed using FS Universal SYBR Green Master ROX (Roche, SWISS) and synthesized cDNA templates. PCR amplification was carried out under the following cycling conditions: initial denaturation at 95 °C for 5 s, followed by 40 cycles of denaturation at 95 °C for 5 s and annealing/extension at 60 °C for 30 s. Each reaction was run in triplicate, and rat GAPDH or human GAPDH was used as an internal control for normalization. The sequences of the primers used for the qRT‒PCR analysis are listed in Table 1, Table 2.

**Table 1:** Rat primer sequences for RT‒PCR *in vitro*.

Gene	Forward	Reverse
*Adiponectin*	GACTGCCACTAATTCAGAGC	CTCATGGGGATAACACTCAG
*Adipor1*	AGGTGGTAAGATTCGGGTCA	TGAATGCCAAGCGGTCTGTA
*Adipor2*	CCACAACCTTGCTTCATCTA	GATACTGAGGGGTGGCAAAC
*Acsl4*	GAGTCCAGACCTTCCCTGTCA	ACATTGCTCAAGACACTTGCC
*Elovl5*	GAGTCTCACCCTTCATCCCTT	AGCAAAACTGGTGAACCCTCC
*Cd36*	CATTTGCAGGTCTATCTACG	CAATGTCTAGCACACCATAAG
*Slc27a4*	CTGAGCTGCACAAAACAGGTC	AGATTAGGCAGAGGGCTCAGA
*Hadha*	TGTCCTTGTGCAGGTTACTGT	CGGGGTAAACTGTGTCCCAT
*Gapdh*	AAACCCGTACAGCGTCCTTC	CCCCACCATCCAGTTCCTAT

**Table 2:** Human primer sequences for RT‒PCR *in vitro*.

Gene	Forward	Reverse
*ADIPONECTIN*	GCTGGAGTTCAGTGGTGTGA	ACCAACCTGACGAATGTGGT
*ADIPOR1*	GATGTAGCGCGGGGGAC	AGCTTCAGCTTGGGGAAAGG
*ADIPOR2*	AGACACGCGGATCAACTCAC	CTGCACCCCAATCGGTTTTC
*ACSL4*	AACCATTCCAAGGCATTCCA	ACCCCAAATGTCTACCAACAGA
*EVOVL5*	ATCAGCTCCAGTGTTCAACC	GTGTGGTAGCCGGTAGGTTT
*CD36*	GACATGTCTAGCCACTGATCATTTT	CAACTTTGGCACAAGTGCTTT
*SLC27A4*	AAGGAACTGCCCCTGTATGC	CTGCTCACCTCTACACAGGC
*HADHA*	CCACATCCCCTTTGACTCCA	GGGTCTTCTGCTTTGCTGAGT
*PPARα*	CTGTGGAGGTGAGTGGTTGA	ATGTGAGTGTCCCCTCCACT
*CPT1A*	AGAGGAACCGAAAGCCTGTG	TGTAAATCCGCCCTTGGCTT
*GAPDH*	CTGGTGGCTGGCTCAGAAAA	TGGTCCAGGGGTCTTACTCC

### 2.10 Protein isolation and western blot analysis

The samples were lysed in lysis buffer (Sigma–Aldrich, USA) supplemented with a phosphatase inhibitor (AG Scientific, USA) and a protease inhibitor cocktail (Roche, SWISS). Protein lysates were separated by sodium dodecyl sulfate‒polyacrylamide gel electrophoresis (SDS‒PAGE) and transferred to PVDF membranes (Bio-Rad, USA). The primary antibodies used were as follows: anti-PPARα (1:2000, NB300-537, Novus Biologicals, USA), anti-CPT1A (1:2000, ab128568, abcam, USA), anti-phospho-acetyl-COA (1:1000, 3661S, Cell Signaling Technology, USA), anti-acetyl-CoA (1:1000, 3662S, Cell Signaling Technology, UK), anti-COX IV (1:1000, 4844S, Cell Signaling Technology), anti-Lamp2a (1:2000, 51–2200, Invitrogen), anti-PLIN2 (1:1000, PQ1-16972, Invitrogen), anti-ATGL (1:1000, 2138S, Cell Signaling Technology), anti-HSL (1:1000, 4107S, Cell Signaling Technology), anti-ERα (1:2000, MA1-310, Invitrogen), anti-CYP17A1 (1:1000, bms-54306R, Bioss), anti-CYP19A1 (1:2000, PA1-16532, Invitrogen), anti-StAR (1:2000; 8449S; Cell Signaling Technology), ADIPONECTIN RECEPTOR 1 (1:2000, MA5-32249, Invitrogen), ADIPONECTIN RECEPTOR 2 (1:2000, bs-0611R, Bioss) and anti-GAPDH (1:2000; GTX100118; GeneTex) antibodies. The following secondary antibodies were used: anti-HRP-conjugated mouse IgG (1:5000; 7076S; Cell Signaling Technology, USA) and anti-HRP-conjugated rabbit IgG (1:5000; 7074S; Cell Signaling Technology, USA). Each band was subjected to chemiluminescence detection using ECL reagent (Bio-Rad, USA) and quantified using ImageJ software (NIH, USA).

### 2.11 Hematoxylin and eosin (H&E) staining to count follicles

The ovary tissues were fixed in 10% neutral buffered formalin (BBC, USA), embedded in paraffin, and serially sectioned at a thickness of 4 μm. The sections were deparaffinized with xylene and a graded ethanol series in a dry oven at 60 °C and rehydrated in distilled water. Hematoxylin and eosin (H&E) staining was performed by immersing the sections in Harris hematoxylin (Leica Biosystems, Germany) for 7 min, followed by counterstaining with alcoholic eosin Y (BIOGNOST, Croatia). After dehydration and mounting, whole-slide digital scans of the stained sections were acquired using a 3DHISTECH scanner (The Digital Pathology Company, Hungary). To quantify folliculogenesis, follicles in the sections were counted at 100 μm intervals across the entire ovary to prevent double counting. Only follicles with clearly visible oocyte nuclei were counted. Follicles were categorized as primordial (oocyte surrounded by a single layer of flattened granulosa cells), primary (one layer of cuboidal granulosa cells), secondary (multiple layers of granulosa cells without an antrum), or antral (presence of a fluid-filled antral cavity). Counting was performed manually by three blind independent observers, and the total number of follicles in each category per ovary was recorded [25].

### 2.12 Nile red staining

For *in vitro* analysis, theca cells and KGN cells were harvested, seeded onto glass coverslips, and fixed with 4% paraformaldehyde. After being washed with PBS, the cells were stained with 0.5 μg/mL Nile red under the same conditions as described for the tissue sections and thoroughly washed with PBS.

### 2.13 Immunofluorescence staining

For immunofluorescence analysis, fixed cells were blocked with DAKO blocking solution for 1 h at room temperature, followed by overnight incubation at 4 °C with primary antibodies against CPT1A (1:200; PA5-76788; Invitrogen, USA) and PLIN2 (1:1000; PQ1-16972; Invitrogen, USA). The next day, the cells were washed three times with PBS (5 min each) and incubated with the appropriate secondary antibody for 1 h at room temperature, followed by three final PBS washes. Finally, the slides were mounted using VECTASHIELD mounting medium containing DAPI (Vector Laboratories, USA) for nuclear staining. Fluorescent signals were visualized using a fluorescence microscope, and representative images were acquired. Quantification of fluorescence intensity was performed using ImageJ software (version 1.54g, Java 1.8.0_345, 64-bit).

### 2.14 BODIPY staining

KGN cells were fixed with 4% paraformaldehyde for 10 min. The samples were incubated with a 10 μg/ml BODIPY® 505/515 (Thermo Fisher Scientific, USA) solution for 30 min at 37 °C. The samples were rinsed with 1 × PBS and mounted using mounting medium containing DAPI (VECTASHIELD, USA). A fluorescence microscope (Zeiss Axiocam 506 color, Germany) was used to observe the cells at 40 × magnification. All parts of each slide were observed, and representative images were captured.

### 2.15 Immunohistochemical staining

For antigen retrieval, deparaffinized tissue sections were treated with EDTA buffer (eLbio, Republic of Korea) according to the manufacturer’s instructions. Endogenous peroxidase activity was blocked using 3% hydrogen peroxide at room temperature for 10 min. Next, the slides were incubated overnight at 4 °C with primary antibodies against CYP17A1 (1:250; ab231794; Abcam) and CYP19A1 (1:500; PA1-16532; Invitrogen). On the following day, after thorough washing to remove unbound primary antibodies, the tissues were incubated at room temperature for 1 h with Dako Real EnVision™ HRP-conjugated rabbit/mouse secondary antibody (Dako, USA). Immunoreactive signals were visualized using a 3,3′-diaminobenzidine (DAB) substrate kit (Dako), followed by counterstaining with hematoxylin. After staining, the slides were rinsed, mounted and analyzed using a 3DHISTECH digital pathology system (The Digital Pathology Company).

### 2.16 Enzyme-linked immunosorbent assays (ELISAs)

All blood samples were collected from the aorta of rats using aortic puncture. After centrifugation, the serum was isolated, and the separated serum samples were stored at −80 °C until use. Levels of rat adiponectin (ab108784; Abcam), LH (MBS764675; MyBioSource), FSH (MBS20211901; MyBioSource), testosterone (MBS702057; MyBioSource), estrogen (MBS2607338; MyBioSource), AMH (MBS701712; MyBioSource), and StAR (MBS729454; MyBioSource) in serum were measured using commercial ELISA kits following the manufacturers’ protocols. Briefly, equal volumes of serum samples were added to antibody-coated ELISA plates, followed by incubation with horseradish peroxidase (HRP)-conjugated secondary antibodies at 37 °C. Substrate solution was then added for incubation in the dark for color development. The enzymatic activity was detected and quantified using a microplate reader (BioTek). Each sample was assayed in triplicate, and the results are presented as relative values. In addition, supernatants collected from cultured KGN cells were analyzed using the same ELISA procedure to assess the secretion levels of the target proteins human adiponectin (ab99968; Abcam) under *in vitro* conditions.

### 2.17 Statistical analysis

The data are presented as the mean ± standard deviation (SD). To compare two independent groups, we employed the nonparametric Mann–Whitney U test (also referred to as the Wilcoxon rank-sum test). This test was chosen because of its suitability for non-normally distributed data and its reliance on rank-based comparisons, making it appropriate for both ordinal and continuous data that do not meet the assumption of normality. The analysis was conducted using wilcox.test. For comparisons involving more than two groups, the Kruskal–Wallis H test, a nonparametric alternative to one-way ANOVA, was applied to assess whether there were statistically significant differences between group medians. The analysis was conducted using kruskal.test. If the Kruskal–Wallis test indicated a statistically significant difference (*p* < 0.05), pairwise comparisons between groups were conducted using the Conover–Iman post hoc test. This correction controls the false discovery rate (FDR), minimizing the risk of false positives in multiple hypothesis testing. A *p* value of less than 0.05 for all tests was considered to indicate statistical significance.

## 3 Results

### 3.1 PDMSC I.V. Administration ameliorates systemic metabolic homeostasis in TAA-induced model

**Figure 1:**
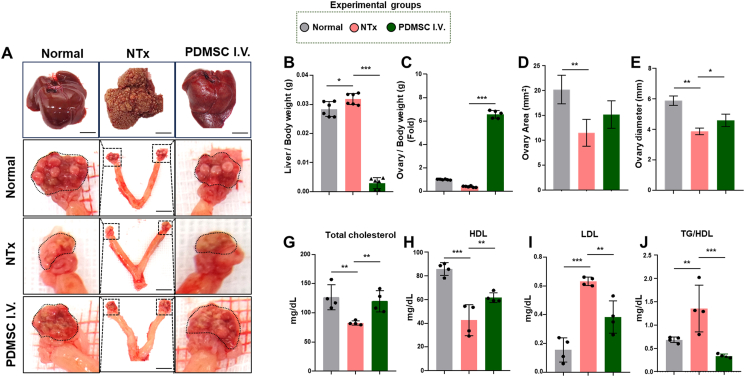
**PDMSC administration restores systemic metabolic homeostasis in TAA-induced rats.** (A–C) Liver/ovary-to-body weight ratios (*n* = 5–6). (D, E) Ovarian surface area and diameter (*n* = 5–6). (G–J) Serum levels of total cholesterol, HDL, LDL, and TG/HDL ratio analyzed by blood chemistry (*n* = 4). The Mann–Whitney U test was used to compare two independent groups, as it is suitable for nonnormally distributed data. A *p* value < 0.05 was considered to indicate statistical significance; ∗*p* < 0.05; ∗∗*p* < 0.01; ∗∗∗*p* < 0.001 vs. the other groups.

In the TAA-induced metabolic dysfunction model, liver tissues exhibited extensive nodular fibrosis indicating severe hepatic injury, while the ovaries showed evident atrophic changes. In contrast, rats transplanted with PDMSCs displayed markedly improved gross morphology of both the liver and ovaries, which resembled those of normal controls (Figure 1A). The liver-to-body weight ratio was significantly elevated in the NTx group, reflecting hepatomegaly, but this parameter was restored in the PDMSC I.V. (∗*p* < 0.001; Figure 1B). The decreases in the ovarian weight and size indices induced by TAA were partially reversed after PDMSC I.V. (∗*p* < 0.001; Figure 1C). These results indicate that PDMSC I.V significantly restored the altered liver and ovarian weight ratios induced by TAA injury. Additionally, the ovarian surface area and diameter, both of which decreased in the NTx group, were partially restored in the PDMSC I.V., indicating improved ovarian tissue structure and integrity (Figure 1D, E). These findings suggest that PDMSC I.V. may increase the weight of ovarian tissue. Serum lipid analysis showed that TAA-induced rats exhibited decreased HDL levels and elevated TG/HDL and TC levels. These dyslipidemia profiles were significantly ameliorated in the PDMSC I.V., indicating improved systemic lipid metabolism following PDMSC therapy (∗*p* < 0.01; Figure 1G–J).

Collectively, these data suggest that PDMSC administration has potent systemic effects on restoring hepatic and ovarian morphology and normalizing lipid profiles in a TAA-induced model of metabolic dysfunction.

### 3.2 PDMSCs attenuate lipid toxicity and induce metabolic remodeling via ADIPONECTIN-fatty acid driven signaling pathway

**Figure 2:**
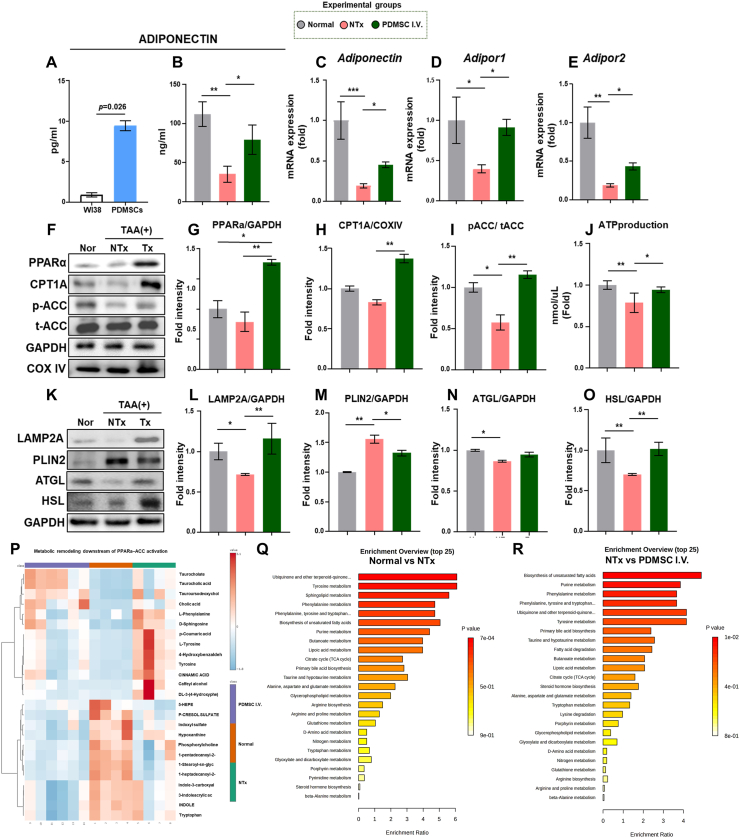
**PDMSCs mitigate lipid toxicity via the ADIPONECTIN-Fatty acid driven axis.** (A, B) Adiponectin levels in PDMSC supernatant and rat serum by ELISA. (C–E) Ovarian mRNA levels of *Adiponectin*, *Adipor1*, and *Adipor2* by qRT-PCR. (F–I) Representative western blots and quantification of PPARα, CPT1A, and p-ACC in ovaries. (J) Serum ATP levels. (K–O) Western blot analysis of Lamp2a, PLIN2, ATGL, and HSL. (P–R) Serum metabolomic heatmap and pathway enrichment analysis. The data are shown as the mean ± SD. The Mann–Whitney U test was used to compare two independent groups, as it is suitable for nonnormally distributed data. A *p* value < 0.05 was considered to indicate statistical significance; ∗*p* < 0.05; ∗∗*p* < 0.01; ∗∗∗*p* < 0.001 vs. the other groups.

ADIPONECTIN, a key adipocytokine, is a fundamental regulator of systemic glucose and lipid homeostasis [26]. PDMSCs secreted significantly higher levels of adiponectin compared with WI38 fibroblasts, supporting a paracrine mechanism within the ovarian microenvironment (∗*p* = 0.026; Figure 2A). In vivo, PDMSC I.V. effectively restored reduced serum adiponectin levels in the NTx group and significantly upregulated ovarian mRNA expression of *Adiponectin*, *Adipor1*, and *Adipor2* (∗*p* < 0.05; Figure 2B–E). Western blot analysis confirmed that the TAA-induced decrease in PPARα, CPT1A, and p-ACC was robustly reversed by PDMSC I.V. (Figure 2F–I). Notably, PDMSCs increased CPT1A protein abundance within isolated mitochondrial fractions, directly enhancing mitochondrial fatty acid oxidation (FAO) capacity [27] (∗*p* < 0.01; Figure 2H). This metabolic shift was accompanied by restored serum ATP levels and a significant modulation of lipid droplet-associated proteins [28], including reduced PLIN2 and increased LAMP2A and ATGL (Figure 2J–O). Furthermore, untargeted metabolomic profiling revealed that PDMSC I.V. shifted the dysregulated metabolic signature of the NTx group toward the normal profile, particularly normalizing sphingolipid and glycerophospholipid metabolism (Figure 2P–R). These results demonstrate that the ADIPONECTIN-Fatty acid driven axis acts as a master switch to induce global metabolic remodeling and clear toxic lipids.

### 3.3 PDMSC transplantation restores ovarian reserves and steroidogenesis in a TAA-induced metabolic syndrome model

**Figure 3:**
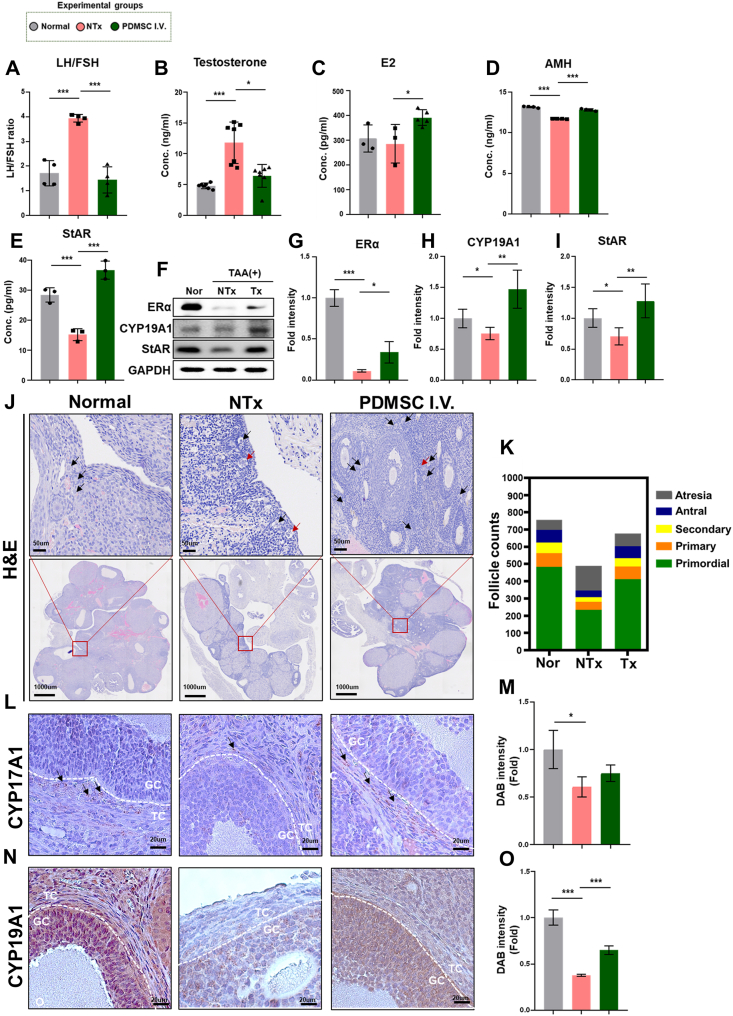
**PDMSC transplantation restores ovarian reserves and steroidogenesis.** (A–E) Serum levels of LH/FSH ratio, testosterone, E2, AMH, and StAR by ELISA (*n* = 3–8). (F–I) Ovarian protein expression of ERα, CYP19A1, and StAR. (J) H&E staining of follicles. (K) Follicle counts by developmental stage. (L–O) IHC staining for CYP17A1 and CYP19A1. Scale bar: 2000 μm; 50, 20 μm magnification: 1.4×. The data are shown as the mean ± SD. The Mann–Whitney U test was used to compare two independent groups, as it is suitable for nonnormally distributed data. A *p* value < 0.05 was considered to indicate statistical significance; ∗*p* < 0.05; ∗∗*p* < 0.01; ∗∗∗*p* < 0.001 vs. the other groups.

TAA-induced metabolic dysfunction resulted in severe endocrine disruption, characterized by an increased LH/FSH ratio and testosterone levels alongside decreased estradiol and AMH (∗*p* < 0.05; Figure 3A–D). Following PDMSC I.V., serum StAR levels and ovarian protein expression of ERα, CYP19A1, and StAR were significantly restored compared to the NTx group (∗*p* < 0.05; Figure 3E–I). Histological assessment via H&E staining confirmed that PDMSCs protected the follicle pool from TAA-induced damage, significantly reducing follicular atresia and preserving the primordial follicle population (∗*p* < 0.001; Figure 3J–K, see Table 3). To further investigate functional recovery, immunohistochemical staining demonstrated that PDMSCs restored the layer-specific localization of CYP17A1 in theca cells and CYP19A1 in granulosa cells [29] (∗*p* < 0.001; Figure 3L–O). These findings suggest that PDMSCs rejuvenate the ovarian microenvironment by modulating steroidogenic pathways in a cell-specific manner.

**Table 3:** Ovarian follicle counts in experimental groups.

	Primordial	Primary	Secondary	Antral	Atresia
Nor (*n* = 6)	484.72 ± 21.37	78.89 ± 12.40	62.17 ± 12.42	73.13 ± 13.10	57.42 ± 7.46
NTx (*n* = 8)	234.42 ± 19.62∗∗∗	46.16 ± 4.64∗	26.75 ± 6.25∗	39.53 ± 3.75∗	142.13 ± 14.22∗∗∗
PDMSC I.V. (*n* = 6)	412.21 ± 21.08∗∗∗	73.00 ± 10.79∗	48.71 ± 6.32∗	68.96 ± 5.92∗	75.04 ± 8.56∗∗∗

∗Nor vs. NTx (*p* < 0.05); ∗∗: Nor vs. NTx (*p* < 0.01); ∗∗∗: Nor vs. NTx (*p* < 0.001).

∗NTx vs. PDMSC I.V (*p* < 0.05); ∗∗: NTx vs. PDMSC I.V (*p* < 0.01); ∗∗∗: NTx vs. PDMSC I.V (*p* < 0.001).

### 3.4 PDMSCs promote mitochondrial fatty acid oxidation and lipid homeostasis in TAA-injured theca cells

**Figure 4:**
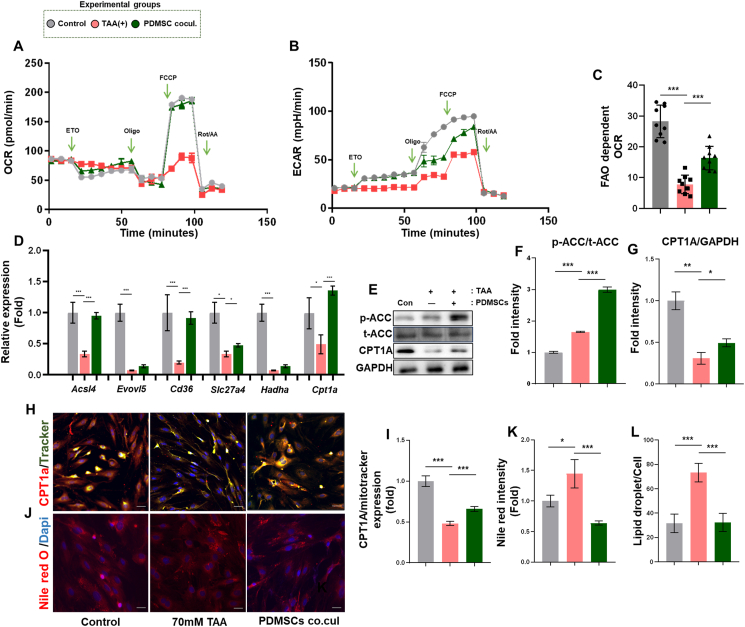
**PDMSCs enhance mitochondrial FAO and lipid homeostasis in primary theca cells.** (A, B) OCR and ECAR assays. (C) FAO-dependent OCR. (D) mRNA levels of FAO markers. (E–G) Protein levels of p-ACC and CPT1A. (H, I) CPT1A/MitoTracker immunofluorescence. (J–L) Nile red staining and lipid droplet quantification. *n* = 3; ∗*p* < 0.05, ∗∗*p* < 0.01, ∗∗∗*p* < 0.001 vs. other groups. (Con: Control; TAA: Thioacetamide; PDMSC cocul.: cocultivation).

To evaluate the metabolic rescue of primary theca cells, we analyzed mitochondrial respiration using Seahorse XF technology. TAA treatment significantly reduced basal respiration, ATP production, and maximal respiration, indicating severe mitochondrial dysfunction, while PDMSC coculture robustly improved these parameters (Figure 4A–B). Specifically, FAO-dependent respiration, measured by etomoxir-sensitive OCR, was significantly restored in the PDMSC group compared to the TAA group (∗*p* < 0.001; Figure 4C). This functional recovery was supported by the significant upregulation of FAO-associated markers, including *Acsl4*, *Elovl5*, *Cd36*, *Slc27a4*, *Hadha*, and *Cpt1a* (∗*p* < 0.05; Figure 4D). Furthermore, western blot and immunofluorescence analyses confirmed increased p-ACC and CPT1A protein levels, which were accompanied by a marked reduction in lipid droplet accumulation as shown by Nile red staining (∗*p* < 0.001; Figure 4E–L). These findings indicate that PDMSCs restore impaired fatty acid utilization and alleviate the intracellular lipid burden in injured theca cells.

### 3.5 PDMSCs promote mitochondrial fatty acid oxidation and lipid homeostasis in TAA-injured granulosa cell

**Figure 5:**
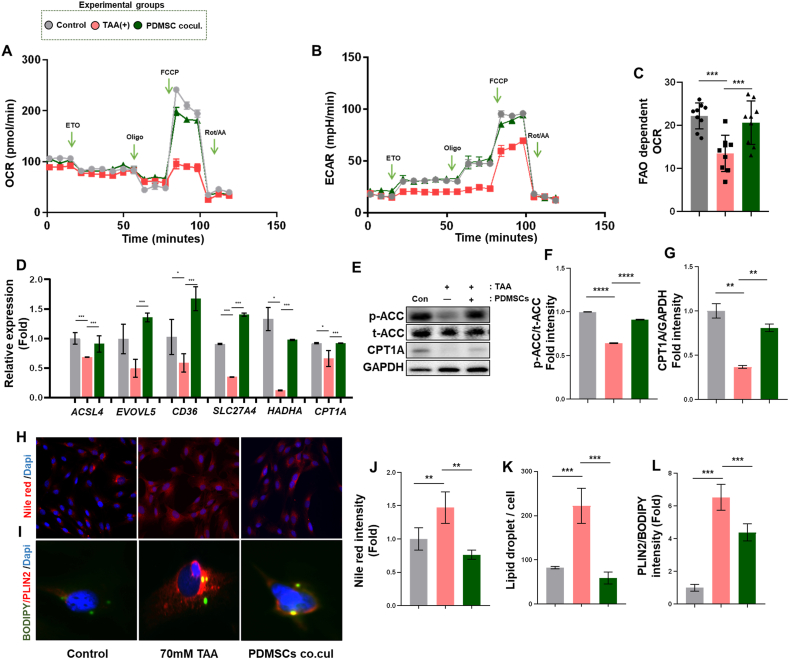
**PDMSCs improve mitochondrial FAO and lipid balance in granulosa cells.** (A–C) OCR, ECAR, and FAO-dependent OCR assays. (D) FAO-related gene expression. (E–G) p-ACC and CPT1A protein levels. (H–K) Nile red staining and lipid droplet analysis. (I, L) PLIN2/BODIPY immunofluorescence. *n* = 3; ∗*p* < 0.05, ∗∗*p* < 0.01, ∗∗∗*p* < 0.001.

Consistent with the findings in theca cells, Seahorse XF analysis of TAA-injured granulosa cells (GCs) revealed severely reduced basal OCR and ATP-linked respiration, which were significantly restored by PDMSC coculture (Figure 5A–B). The PDMSC group exhibited a significant increase in FAO-dependent respiration, supporting the reactivation of mitochondrial fatty acid utilization pathways (∗*p* < 0.001; Figure 5C). Gene expression analysis further showed that critical FAO-related markers such as *ACSL4, ELOVL5, CD36, SLC27A4, HADHA,* and *CPT1A* were significantly upregulated in cells cocultured with PDMSCs (∗*p* < 0.001; Figure 5D). Western blot analysis validated these results, showing increased ACC phosphorylation and CPT1A protein expression (Figure 5E–G). Additionally, Nile red and BODIPY/PLIN2 double staining demonstrated a significant reduction in lipid droplet accumulation, suggesting that PDMSCs enhance mitochondrial FAO capacity to clear toxic lipids and restore lipid homeostasis in damaged GCs (Figure 5H–L).

### 3.6 Pharmacological inhibition of CPT1A by etomoxir abrogates the restorative effect of PDMSCs on ovarian steroidogenic function

**Figure 6:**
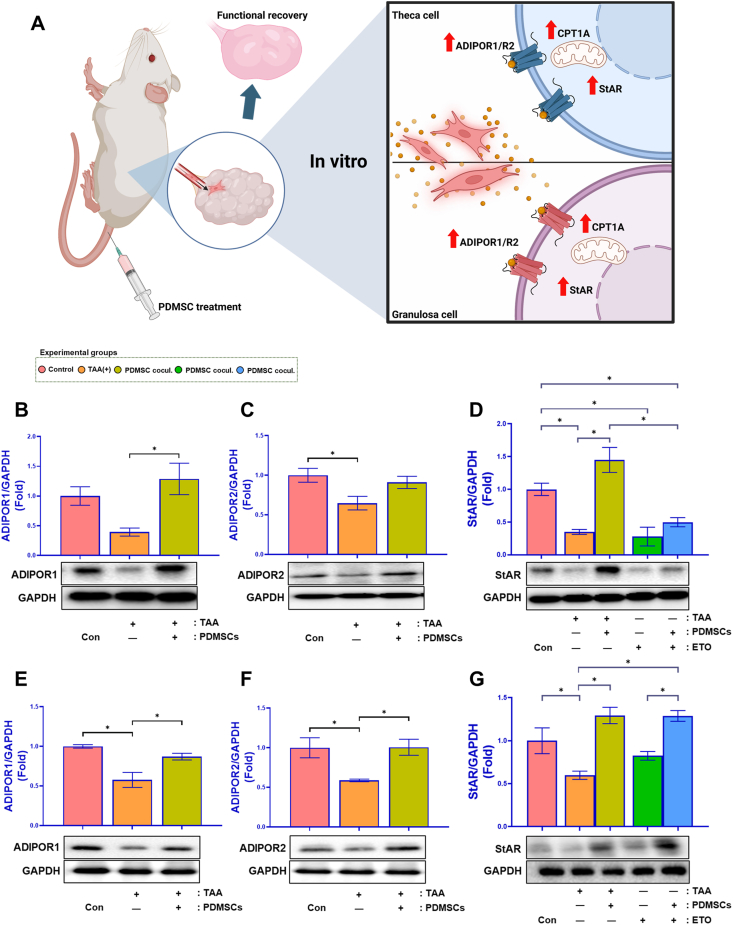
**Pharmacological inhibition of CPT1A by etomoxir abrogates the restorative effect of PDMSCs on ovarian steroidogenic function.** (A) Scheme illustration of ADIPOR1 and ADIPOR2 signaling pathways in isolated theca and granulosa cells following TAA treatment and PDMSC co-culture. (B, C) Quantification of western analysis of ADIPOR1 and ADIPOR2 in Theca cells. (D) Quantitative analysis of StAR in Theca cells. (E, F) Quantification of ADIPOR1 and ADIPOR2 in Granulosa cells. (G) Quantitative analysis of StAR expressions in Granulosa cells. Data are presented as the mean ± SD of three independent experiments. GAPDH was used as an internal loading control. ∗*p* < 0.05 indicates statistical significance between groups. (Con: Control; TAA: Thioacetamide; PDMSCs: Placenta-derived mesenchymal stem cells; ETO: Etomoxir).

To verify the functional necessity of the ADIPOR–Fatty acid driven axis, we established an *in vitro* model using TCs and GCs treated with TAA to induce metabolic damage, followed by co-culture with PDMSCs. First, we confirmed that TAA treatment significantly suppressed the expression of ADIPOR1 and ADIPOR2 in both cell types, while co-culture with PDMSCs effectively restored their protein levels (Figure 6B–C, E–F). The most critical evidence for the mechanism was obtained by blocking CPT1A activity with its specific inhibitor, Etomoxir (ETO). In the TAA-injured groups, PDMSC co-culture robustly increased the expression of StAR, a key steroidogenic enzyme, in both TCs and GCs. However, the pharmacological inhibition of CPT1A by ETO treatment completely abolished this PDMSC-mediated rescue of StAR expression (Figure 6D,G). These findings demonstrate that CPT1A-dependent FAO is the indispensable pathway through which PDMSCs restore steroidogenic function in follicular cells under metabolic stress, as the inhibition of this pathway directly prevents the recovery of StAR expression even in the presence of PDMSCs.

### 3.7 ADIPOR1/ADIPOR2 are essential mediators of the ADIPONECTIN/fatty acid driven axis in GC fatty acid oxidation

**Figure 7:**
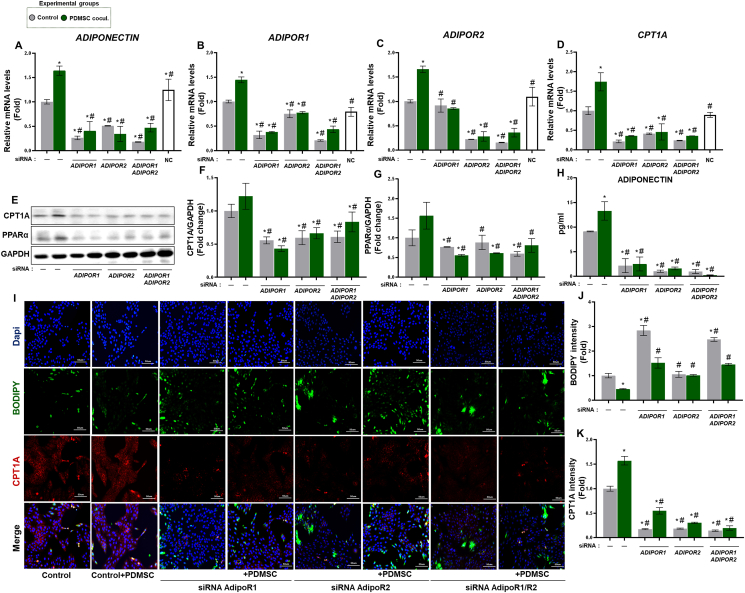
**ADIPOR1/2 are essential for the ADIPONECTIN/Fatty acid driven axis in GCs.** (A–D) mRNA expression of *ADIPONECTIN*, *ADIPOR1/2*, and *CPT1A*. (E–G) Western blots of CPT1A and PPARα. (H) Secreted adiponectin by ELISA. (I–K) BODIPY and CPT1A immunofluorescence and quantification. *n* = 3; ∗*p* < 0.05 vs. Control; ∗*p* < 0.05 vs. Control + MSC.

To clarify whether PDMSC-derived adiponectin promotes FAO via receptor signaling, we performed siRNA-mediated knockdown of *ADIPOR1* and/or *ADIPOR2* in GCs. Knockdown efficacy was confirmed at the mRNA and protein (Figure 7A–C, H and Fig. S7). Knockdown of either receptor significantly blunted the PDMSC-induced upregulation of downstream FAO regulators, including CPT1A and PPARα, at both mRNA and protein levels (Figure 7D–G). Notably, dual *ADIPOR1/2* inhibition did not show an additive effect, suggesting a non-redundant, receptor-dependent response (Figure 7A–G). Crucially, BODIPY staining revealed that the reduction of lipid droplet accumulation by PDMSCs was markedly attenuated when ADIPOR signaling was blocked (∗*p* < 0.05; Figure 7I–J). Similarly, the activation of CPT1A immunofluorescence was significantly blunted in knockdown conditions compared to control siRNA cells (Figure 7I,K). Collectively, these findings prove that the ADIPOR signaling pathway is an indispensable gateway through which PDMSCs exert their protective metabolic effects in granulosa cells.

## 4 Discussion

In this study, we demonstrated that PDMSC transplantation restores ovarian homeostasis through the activation of the ADIPONECTIN–Fatty acid driven axis in a TAA-induced metabolic ovarian dysfunction model. The TAA-induced model used in this study represents a hepatic injury-driven metabolic dysfunction model, reflecting the emerging concept of the liver–ovary metabolic axis. The pathogenesis of PCOS involves a complex interplay among reproductive, endocrine, and metabolic dysfunctions. Although no single animal model can perfectly recapitulate this heterogeneity, DHT or DHEA induced rodents primarily exhibit hyperandrogenism, whereas non-hormonal metabolic models more accurately reflect systemic metabolic decline [30,31]. Specifically, the TAA-induced model used in this study represents a hepatic injury-driven metabolic dysfunction model characterized by hepatotoxicity, systemic insulin resistance, and chronic inflammation [32,33]. Mechanistically, disturbances in hepatic cholesterol synthesis and fatty acid metabolism can alter systemic lipid availability and impair steroidogenic precursor supply, thereby disrupting ovarian steroidogenesis and follicular development [34]. This inter-organ connection aligns with the emerging concept of a liver–ovary metabolic axis.

Clinical evidence has demonstrated a high prevalence of metabolic dysfunction–associated steatotic liver disease (MASLD) in women with PCOS, supporting the notion that hepatic metabolic impairment contributes directly to reproductive abnormalities [35]. Furthermore, recent Mendelian randomization analyses have provided genetic evidence suggesting that NAFLD is causally associated with an increased risk of PCOS, a link partially mediated through insulin resistance and androgen excess [36].

In this context, disturbances in hepatic cholesterol synthesis and fatty acid metabolism can alter systemic lipid availability and impair the supply of steroidogenic precursors, thereby disrupting ovarian steroidogenesis and follicular development [37,38].

Our findings support the concept that hepatic metabolic injury drives ovarian dysfunction through inter-organ metabolic crosstalk, and PDMSC transplantation effectively reverses this decline. The significant reduction in ovarian diameter and area observed in Figure 1 transcends mere morphological change. Consequently, the restoration of ovarian dimensions following PDMSC transplantation provides robust evidence that these stem cells effectively re-engineered the intraovarian microenvironment to reverse physical waste. A noteworthy finding is that marked increase in insulin resistance despite the absence of significant changes in total body weight (Figs. S6 and S8). This indicates that the TAA model accurately recapitulates non-obese metabolic dysfunction, rather than simple obesity-driven impairment. Given that a substantial proportion of clinical PCOS patients exhibit insulin resistance and ovarian failure despite having a normal BMI [39], our data suggests that PDMSCs possess a versatile therapeutic mechanism capable of simultaneously addressing systemic metabolic imbalance and localized ovarian injury.

In stem cell-based therapy, MSCs influence adiponectin signaling by directly secreting adiponectin or by stimulating endogenous adiponectin expression in damaged tissues [40,41]. Also, models of metabolic and reproductive disease, MSC transplantation has been reported to increase circulating adiponectin levels and improve endocrine balance [42]. Unlike previous studies that often listed the general effects of MSCs [43], this work identifies ADIPONECTIN receptors (ADIPORs) as the central switch for metabolic remodeling within the ovary. In this study, ELISA analysis confirmed that PDMSC-conditioned medium contained significantly higher levels of ADIPONECTIN (∼6,500 pg/ml). While our initial comparisons were made against WI38 fibroblasts (Figure 2A), we further strengthened this finding by comparing PDMSCs with bone marrow-derived MSCs (BMMSCs) in the supplementary data (Fig. S3). While MSC transplantation is known to increase systemic adiponectin and normalize hormonal profiles in other metabolic models [44,45], our data provides a deeper mechanistic link that we suggest that potent paracrine secretion serves as the primary driver for specifically stimulating ADIPOR1/2 within the injured ovarian microenvironment.

At the molecular level, PDMSC transplantation was associated with the activation of ADIPOR signaling, which promotes mitochondrial fatty acid oxidation through the upregulation of CPT1A and PPARα while suppressing lipogenesis and lipid droplet accumulation (Figure 2). These changes are consistent with findings from nonalcoholic steatohepatitis (NASH) models, in which ADIPOR activation enhanced mitochondrial function and reduced lipotoxic stress [46]. Additionally, these protein-level alterations were accompanied by distinct changes in the serum metabolomic profile. Untargeted metabolomic analysis indicated that PDMSC transplantation partially restored pathways involved in lipid and energy metabolism, including sphingolipid, glycerophospholipid, arachidonic acid, and purine metabolism (Figure 2P-R). These changes are not isolated molecular events; rather, they reflect a systemic metabolic remodeling that directly correlates with the enhanced fatty acid utilization observed at the cellular level. Specifically, the normalization of sphingolipid and glycerophospholipid species likely facilitates mitochondrial membrane dynamics and redox homeostasis, reducing oxidative stress-associated follicular damage. In addition to these metabolic improvements, our immunofluorescence analysis demonstrated that PDMSC transplantation significantly improved the expression of LC3B in both theca and granulosa cells, compared to the TAA-treated group (Fig. S1). Given that LC3B is a key executor of autophagy, its restoration indicates that PDMSCs activate cellular quality-control mechanisms to clear damaged organelles and protein aggregates induced by lipotoxic stress.

Furthermore, the significant alterations in systemic ATP levels provide the bioenergetic basis for the observed restoration of follicular development and steroidogenesis. The observed systemic metabolic remodeling was closely consistent with the functional improvements at the ovarian tissue level. PDMSC transplantation successfully attenuated lipid accumulation within the ovarian microenvironment and restored the layer-specific expression of key steroidogenic enzymes, including StAR, CYP17A1, and CYP19A1, thereby re-establishing the endocrine balance disrupted by TAA-induced lipotoxicity (Figure 3). These findings are consistent with those of Sarvestani et al. [45] and Wang et al. [47], who reported that MSC treatment corrected lipid dysregulation and normalized testosterone levels in PCOS-like metabolic models. While previous studies primarily focused on the systemic normalization of testosterone and other hormones, our results extend these observations by providing a deeper mechanistic link, we suggest that adiponectin-mediated signaling serves as the fundamental driver connecting hepatic-driven metabolic correction to the functional restoration of the ovarian steroidogenic machinery.

Importantly, siRNA-mediated knockdown of *ADIPOR1* or *ADIPOR2* abrogated the metabolic and steroidogenic benefits of PDMSCs, indicating that these effects depend on intact adiponectin receptor signaling. Validation of our proposed mechanism was achieved by confirming the siRNA-mediated knockdown of ADIPOR1 and ADIPOR2 at the protein level (Fig. S7). An observation in our mechanistic validation was the differing responses between basal and injury-induced stress conditions. In our initial experiments under normal physiological conditions, we noted that PDMSC co-culture could baseline-upregulate markers such as PPARα, CPT1A, and ADIPONECTIN, even in some siRNA-treated contexts (Figure 7). However, the most critical and definitive evidence emerged from the TAA-induced injury model. While PDMSCs typically trigger a robust recovery in TAA-damaged cells, the siRNA-mediated knockdown of ADIPOR1/2 in the TAA + PDMSC group completely failed to restore these metabolic regulators and lipid accumulation (Figs. S4 and S5). This discrepancy provides a profound mechanistic insight: the therapeutic efficacy of PDMSCs is not merely a redundant paracrine supplement to normal cells but is a stress-specific rescue mechanism. Under the severe lipotoxic stress of TAA, the innate ability of ovarian cells to maintain metabolic homeostasis is compromised, making them strictly dependent on external trophic signals. The fact that recovery was abolished only when the ADIPOR signaling machinery was disrupted in the presence of TAA injury proves that this axis is the gateway through which PDMSCs exert their protective effects. This hierarchical requirement distinguishes PDMSCs from general drugs, positioning them as functional metabolic re-programmers that require intact receptor pathways to counteract systemic injury.

Furthermore, the pharmacological inhibition of CPT1A via ETO revealed that the subsequent recovery of StAR expression is dependent on fatty acid oxidation (Figure 6). This confirms that the restoration of the steroidogenic machinery requires more than just paracrine signaling; it necessitates a functional metabolic switch to secure the bioenergetics required for follicular homeostasis. By establishing causality through receptor silencing and metabolic inhibition under injury conditions, we offer a robust mechanistic framework for PDMSC-based therapy targeting the metabolic components of ovarian failure.

In TAA-induced metabolically impaired ovaries, primordial and antral follicle numbers were significantly reduced, accompanied by increased follicular atresia. PDMSC transplantation partially restored follicular architecture and reduced atresia (Figure 3K). In addition, the expression of BMP15, an oocyte-derived regulator of folliculogenesis and cumulus–oocyte communication, was significantly upregulated following PDMSC treatment (Fig. S2) [48]. These findings suggest that PDMSC therapy may preserve follicular reserve and improve the intraovarian microenvironment.

Despite the significant findings, several challenges and limitations remain to be addressed before clinical translation. First, the therapeutic efficacy of PDMSCs was evaluated at a single dose and time point; further studies are required to determine the optimal dosage and long-term dosing schedules. Second, although the TAA-induced rat model effectively reflects metabolic-reproductive impairment, interspecies differences exist, and the anatomical constraints of the rat ovary may lead to variability in some experimental outcomes. Third, while KGN cells were used to elucidate molecular mechanisms *in vitro*, their status as an immortalized granulosa cell tumor-derived line may not fully recapitulate the physiological characteristics of primary human granulosa cells. Furthermore, practical hurdles such as the efficiency of target homing, potential immunogenicity, long-term safety, and the scalability of clinical-grade cell production must be meticulously investigated.

## 5 Conclusion

In conclusion, this study demonstrates that the transplantation of PDMSCs effectively restores metabolic and reproductive homeostasis in a TAA-induced ovarian dysfunction model. The therapeutic efficacy of PDMSCs is primarily driven by the potent secretion of ADIPONECTIN, which reactivates the ADIPOR1/2–fatty acid driven signaling axis within the ovary. By establishing direct causality through receptor silencing and pharmacological inhibition, we have proven that this axis serves as an indispensable mechanistic bridge linking systemic metabolic improvement with the restoration of follicular steroidogenesis and architecture.

Furthermore, our findings provide robust evidence for the liver–ovary metabolic axis, suggesting that PDMSCs do not merely act as general protective agents but as functional metabolic re-programmers that resolve lipotoxicity through enhanced mitochondrial fatty acid oxidation. These results suggest that PDMSC-based therapy offers a promising and comprehensive strategy for treating the complex interplay of reproductive and metabolic dysregulation in patients with PCOS and related ovarian disorders. By shifting the ovarian microenvironment toward a fatty acid oxidation-dominant state, PDMSCs provide a novel therapeutic framework for addressing metabolic-associated ovarian failure.

## CRediT authorship contribution statement

**Dae Hyun Lee:** Writing – original draft, Investigation, Data curation, Conceptualization. **Jae Young Shin:** Methodology, Data curation. **Hyeri Park:** Methodology, Data curation. **Jin Seok:** Investigation, Data curation. **Kyu Hwan Na:** Formal analysis, Data curation. **Young-Ran Kim:** Investigation, Data curation. **Gi Jin Kim:** Writing – review & editing, Writing – original draft, Funding acquisition, Conceptualization.

## Declaration of competing interest

The authors declare that they have no known competing financial interests or personal relationships that could have appeared to influence the work reported in this paper.

## Data Availability

No data was used for the research described in the article.
