# Supplementary material for: Adiponectin preserves follicles through ADIPOR1/ADIPOR2-driven fatty acid metabolism
Source: Mol Metab. 2026 Jun 10;110:102392. doi: 10.1016/j.molmet.2026.102392 (PMC13311280; doi:10.1016/j.molmet.2026.102392)
Supplement: Multimedia component 1 [file mmc1.pdf]

Supporting Information (without author details)

Adiponectin Preserves Follicles through ADIPOR1/ADIPOR2-Driven Fatty Acid Metabolism

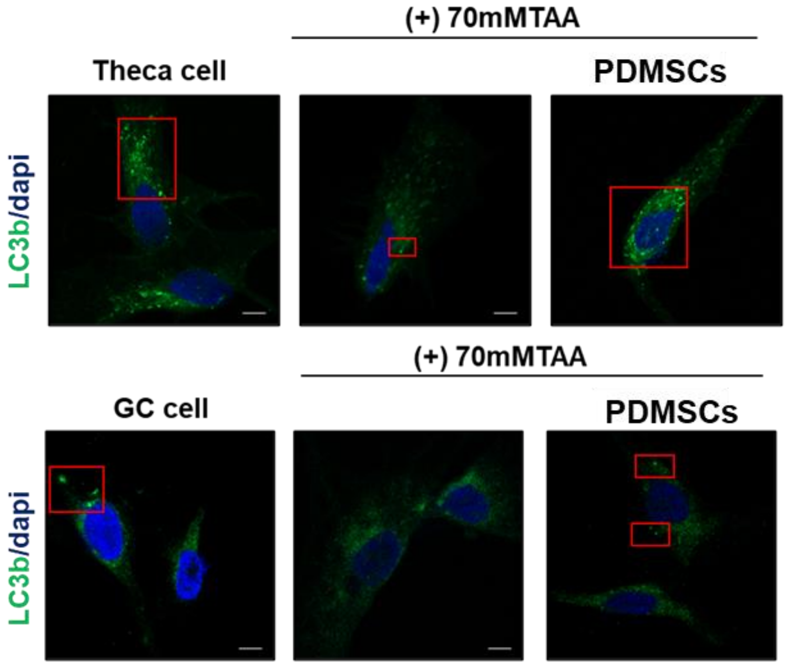

Fig. S1. Analysis of LC3b localization as marker of Autophagy. The localization of LC3b protein expression in ovarian follicles (Theca cell and Granulosa cell) analyzed using immunofluorescence (red box). Scale bar: 50 µm; magnification: 20X.

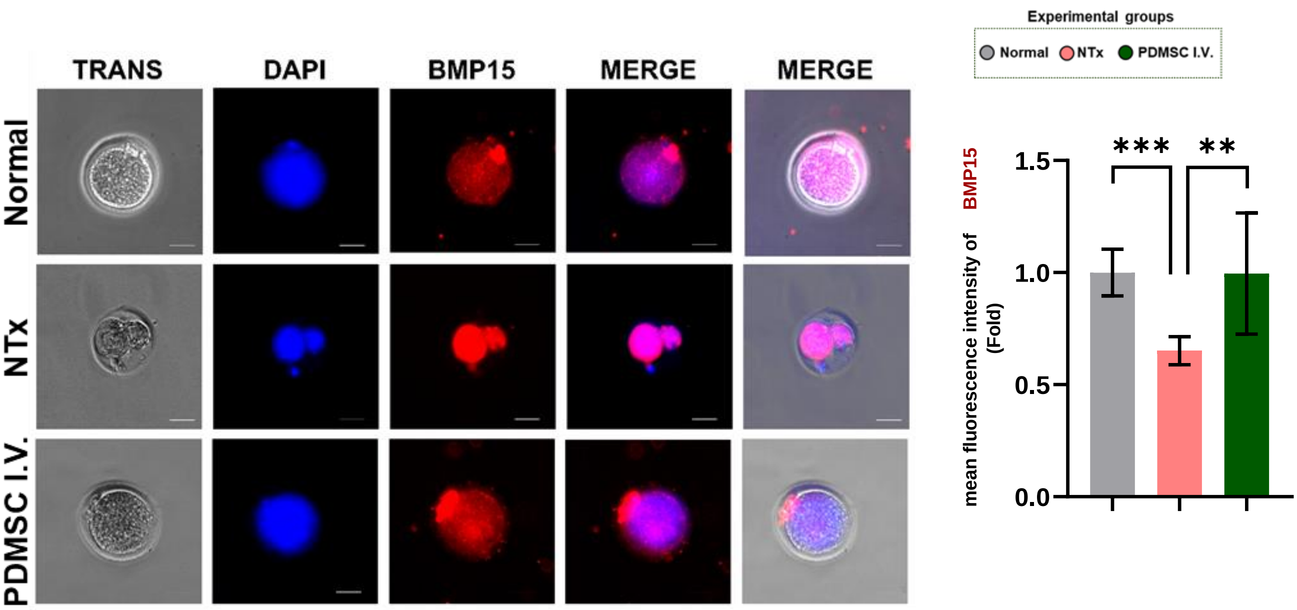

Fig. S2. Analysis of BMP15 localization as marker of follicular development. Rat were superovulated by injecting them with 150 IU of pregnant mare serum gonadotropin (Sigma-Aldrich, St. Louis, MO), followed by 300 IU of human chorionic gonadotropin (hCG; Sigma-Aldrich) 48 h later. Oocytes were collected in M2 (Sigma-Aldrich) medium at 24 h after hCG injection, and cumulus cells were denuded with M2 medium containing 0.1% hyaluronidase (Sigma-Aldrich). The cumulus-free oocytes were then cultured in M2 medium (Sigma-Aldrich) for the experiment. Dispersed cumulus cells were removed by hyaluronidase treatment, diluted in M2 medium, and collected. (***) indicates statistical difference compared to control group (*$p < 0.001$), (**) indicates statistical difference compared to control group (**$p < 0.01$). Data presented as mean ±SD from three independent experiments conducted in triplicate.**

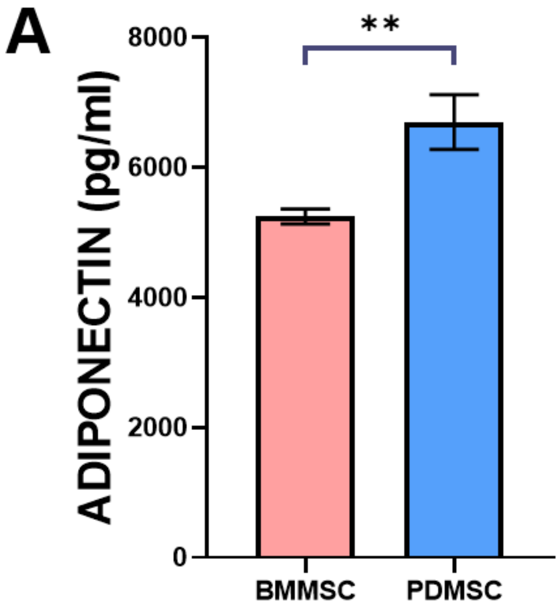

Fig. S3. PDMSCs serve as a paracrine source of adiponectin. (A) Secretion levels of ADIPONECTIN. (*) indicates statistical significance ($p < 0.01$). Data are presented as mean ±SD from three independent experiments conducted in triplicate.**

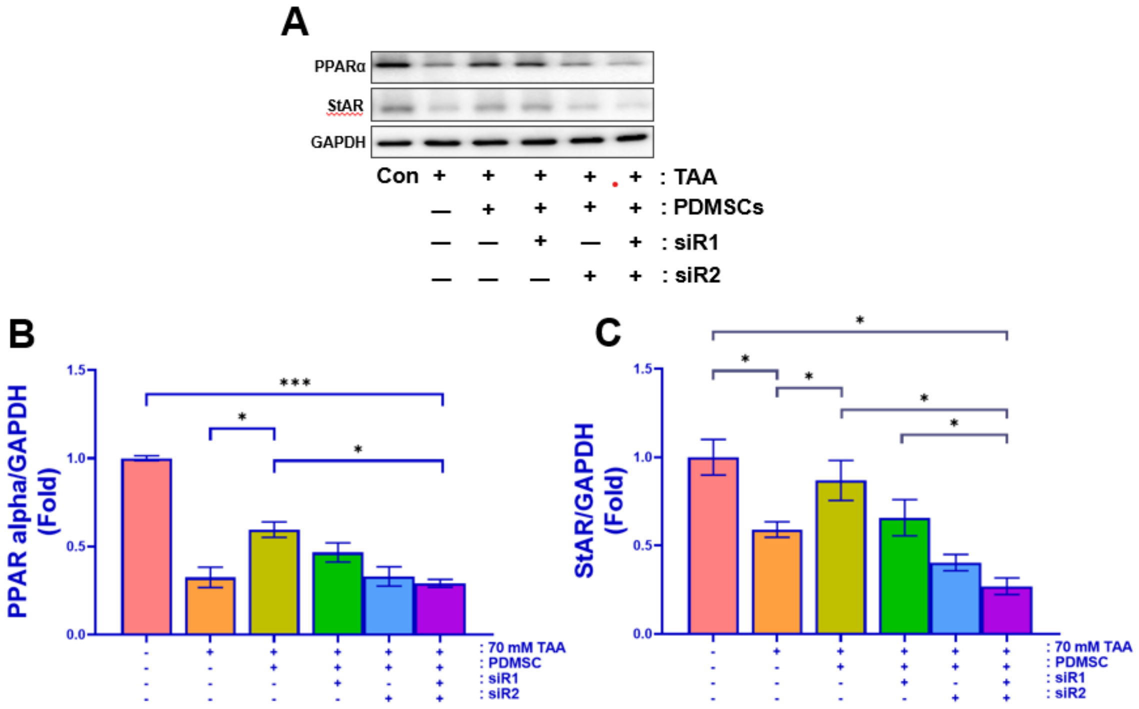

Fig. S4. Abrogation of PDMSC-mediated metabolic rescue by *ADIPOR* silencing. (A) western blot analysis of PPARalpha, StAR protein expression in KGN cells. (B, C) Quantification of protein expression using by image J software. (*) indicates statistical significance (*$p < 0.05$), (**) indicates statistical significance ($p < 0.01$), (***) indicates statistical significance (***$p < 0.001$). Data are presented as mean ±SD from three independent experiments conducted in Triplicate.**

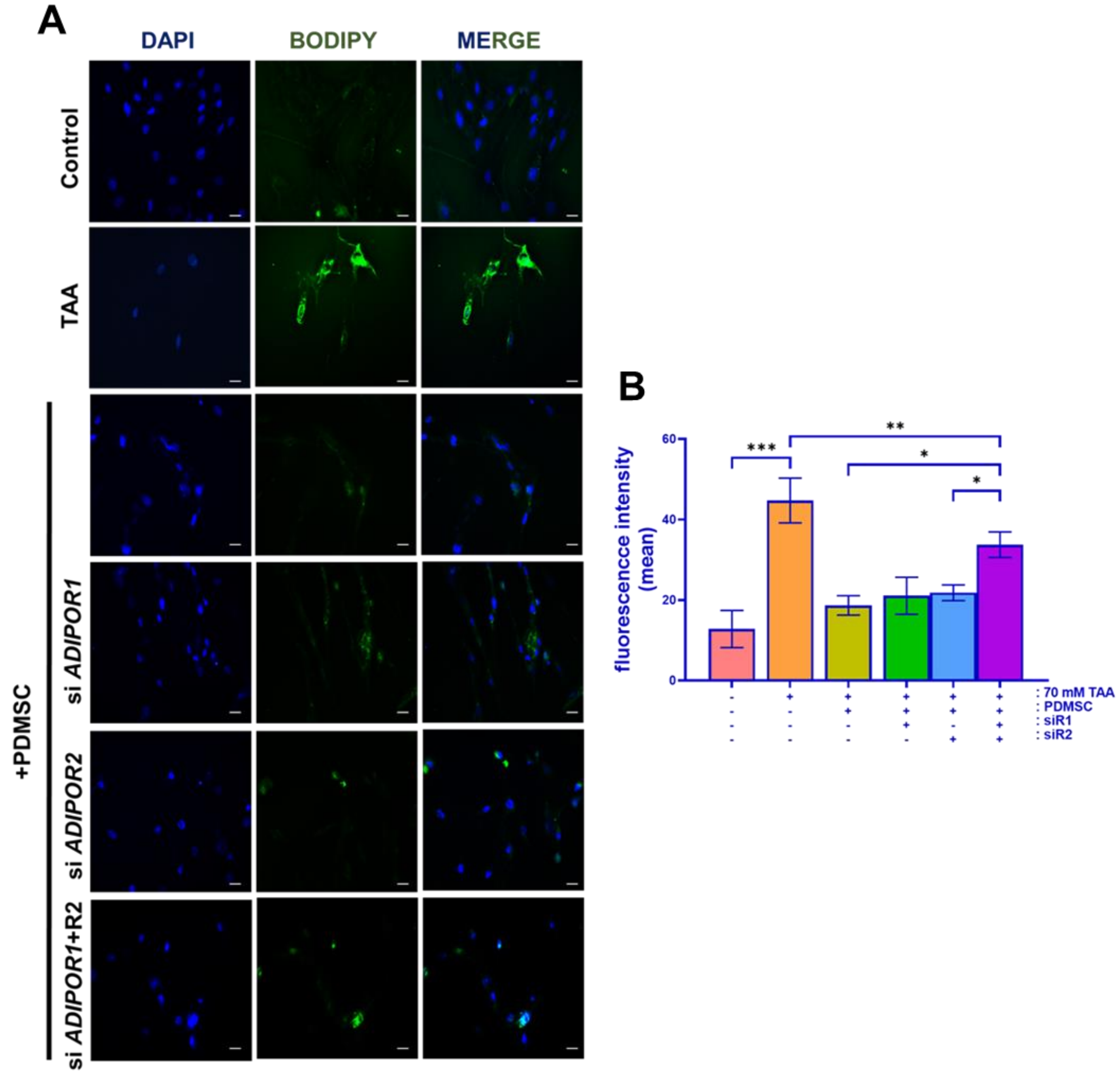

Fig. S5. Re-accumulation of lipids following *ADIPOR* blockade. (A) Representative images of BODIPY staining of KGN cells. (B) Quantificative analysis of BODIPY fluorescence intensity in KGN cells. protein expression using image J software. (*) indicates statistical significance (*p < 0.05), (**) indicates statistical significance (p < 0.01), (***) indicates statistical significance (***p < 0.001). Data are presented as mean ±SD from three independent experiments conducted in Triplicate.**

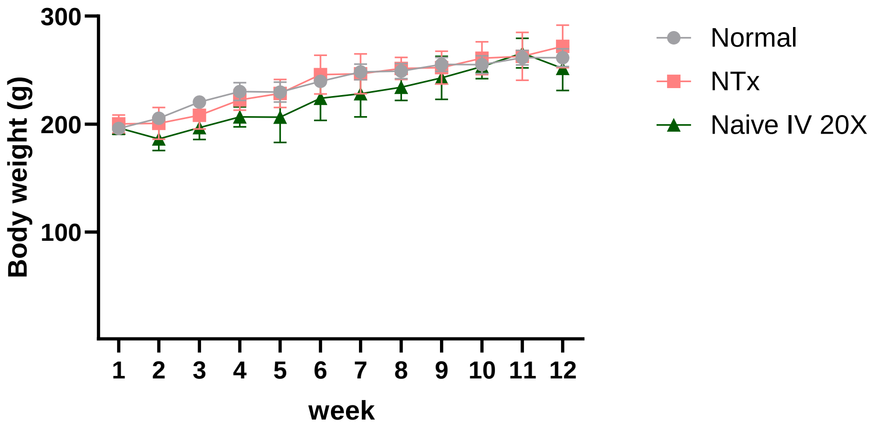

Fig. S6. The body weight of TAA-induced rat model.

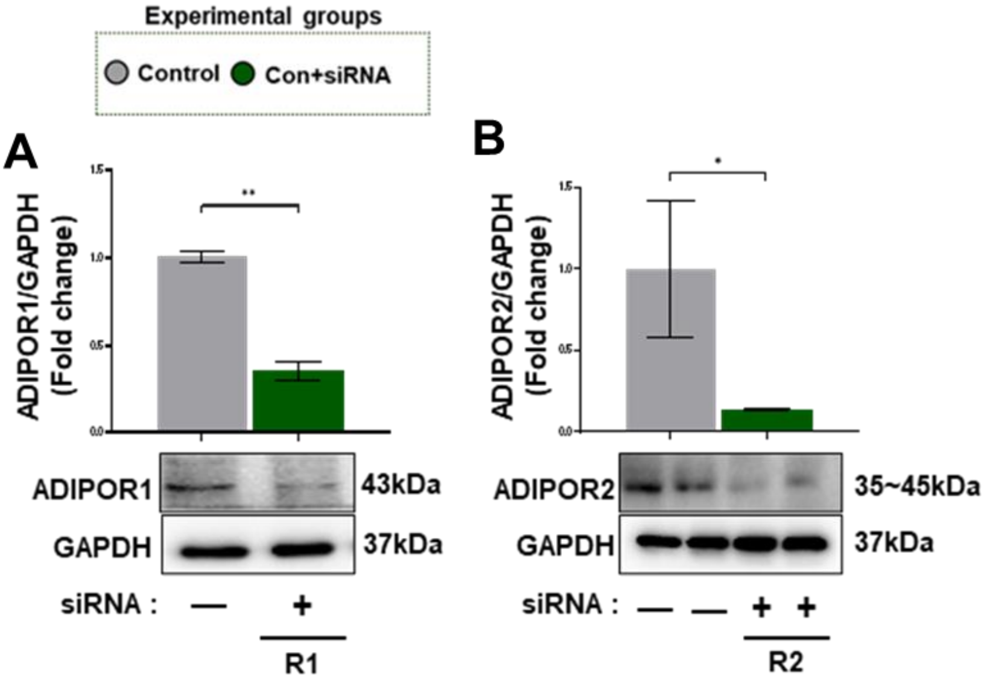

Fig. S7. Validation of siRNA-mediated knockdown efficiency for ADIPOR1 and ADIPOR2. (A-B) western blot of ADIPOR1, ADIPOR2 in granulosa cell. (*) indicates statistical significance (*p < 0.05), (**) indicates statistical significance (p < 0.01). Data are presented as mean ±SD from three independent experiments conducted in Duplicate.**

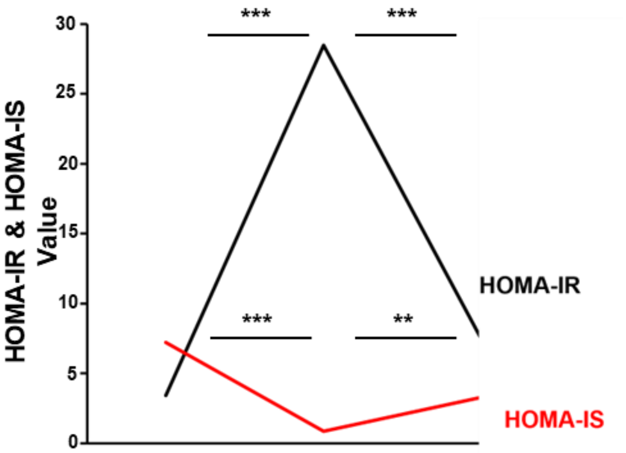

Fig. S8. The body weight of TAA-induced rat model. The HOMA-IR was performed according to the following equation: glucose (mg/dL) × insulin (mU/mL)/405; HOMA-IS was calculated according to the following equation: 1/HOMA-IR, (n=6). (*) indicates statistical significance (p < 0.05), (**) indicates statistical significance (***p < 0.01). Data are presented as mean ±SD from three independent experiments conducted in Duplicate.**
